# Supplementary material for: Comparative Transcriptome Analysis Reveals Common and Developmental Stage-Specific Genes That Respond to Low Nitrogen in Maize Leaves
Source: Plants (Basel). 2022 Jun 10;11(12):1550. doi: 10.3390/plants11121550 (PMC9230787; doi:10.3390/plants11121550)
Supplement: Supplementary file 1 [file plants-11-01550-s001.zip › Supplement material.pdf]

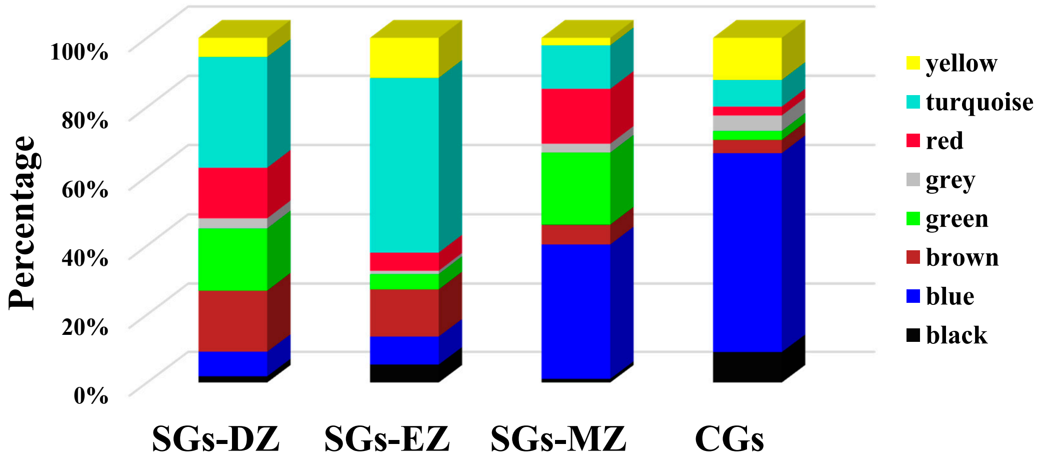

Figure S1. Proportion of genes assigned into different co-expressed modules (CMs) for specific genes (SGs) in different comparisons, namely DZ (SGs-DZ), EZ (SGs-EZ) and MZ (SGs-MZ), as well as common genes (CGs).
